# Body iron status of children and adolescents with transfusion dependent β-thalassaemia: trends of serum ferritin and associations of optimal body iron control

**DOI:** 10.1186/s13104-018-3634-9

**Published:** 2018-08-02

**Authors:** Tharindi Suriapperuma, Ravindu Peiris, Chamila Mettananda, Anuja Premawardhena, Sachith Mettananda

**Affiliations:** 1 Colombo North Teaching Hospital, Ragama, Sri Lanka; 2 Department of Paediatrics, Faculty of Medicine, University of Kelaniya, Thalagolla Road, Ragama, 11010 Sri Lanka; 3 Department of Pharmacology, Faculty of Medicine, University of Kelaniya, Ragama, Sri Lanka; 4 Department of Medicine, Faculty of Medicine, University of Kelaniya, Ragama, Sri Lanka

**Keywords:** Transfusion dependent β-thalassaemia, Serum ferritin, Iron overload, Chelation

## Abstract

**Objective:**

This cross sectional study aims to describe the body iron status, trends of serum ferritin and associations of optimal body iron control in patients aged below 16 years with transfusion dependent β-thalassaemia attending Paediatric and Adolescent Thalassaemia Centres of the Colombo North Teaching Hospital of Sri Lanka.

**Results:**

Out of 54 children, 51% were males and a majority were aged 11–16 years; 83% had β-thalassaemia major while 13% had HbE β-thalassaemia. Mean serum ferritin was 1778(± 1458) µg/l and 29% had optimal serum ferritin (below 1000 µg/l). Trend of mean serum ferritin over time showed gradual decline between 2011 and 2017 and longitudinal trend of individual patients at yearly intervals showed gradual rise until 5 years of age and plateauing thereafter. All except two patients were receiving iron chelator medication of which the most commonly used was oral deferasirox (92%). The most common iron-related complications were short stature (24.1%) and pubertal delay (42.8% of > 14 years). None of the patients had hypothyroidism, hypoparathyroidism or diabetes. Optimal serum ferritin levels were significantly associated with the diagnosis of thalassaemia at a later age (23.6 vs 9.0 months) and higher family income (OR-4.81;95%CI 1.17–19.67) however was not associated with the age of the patient or duration of transfusion.

**Electronic supplementary material:**

The online version of this article (10.1186/s13104-018-3634-9) contains supplementary material, which is available to authorized users.

## Introduction

Thalassaemia is a group of inherited disorders of impaired globin chain synthesis which are characterized by imbalances between α- and β-globin chains in human red blood cells (RBC) [1]. Approximately 70,000 children are born with various forms of thalassaemia worldwide each year [2]. It is considered as one of the most common monogenic disorder in Sri Lanka with approximately 60 new cases being diagnosed every year [3, 4].

Clinical severity of thalassaemia is variable and ranges from mild anaemia that require occasional RBC transfusion (non-transfusion dependent thalassaemia) to severe anaemia which is fatal without regular transfusions (transfusion dependent thalassaemia) [5]. Patients with transfusion dependent thalassaemia require 2–5 weekly RBC transfusions which results in body iron overload. With each unit of packed RBC, 200 mg of iron is infused into the body where there is no effective mechanism to excrete iron [6]. In physiological conditions, iron is bound to a carrier protein transferrin that helps to transport iron into tissues. In situations where there is excess iron, transferrin is saturated resulting in an excess of free iron in the circulation. Free iron is highly reactive and leads to generation of reactive oxygen species causing deleterious effects on cells [7]. This results in organ damage which is the major cause of morbidity and mortality in patients with thalassaemia [8].

Several bio-markers are available to assess the degree of iron overload in thalassaemia patients of which serum ferritin is the most widely used method. Iron chelator medications are initiated in patients with thalassaemia approximately after the tenth RBC transfusions with an aim to maintain serum ferritin below 1000 µg/l. Higher levels of serum ferritin (specially over 2500 µg/l) are associated with iron-related organ failure particularly in the liver and heart. In this study we aim to describe body iron status, trends of serum ferritin and associations of optimal body iron status in patients with transfusion dependent β-thalassaemia.

## Main text

### Materials and methods

A cross sectional study was conducted at the Paediatric and Adolescent Thalassaemia Centres of the Colombo North Teaching Hospital (CNTH), Ragama, Sri Lanka from October to December 2017. CNTH is one of the eight University Hospitals of Sri Lanka and is a tertiary referral centre for Western and North Western Provinces of the country. Thalassaemia centre of CNTH is one of the three main thalassaemia referral centres of Sri Lanka.

All children with transfusion dependent thalassaemia aged 16 years or less attending for blood transfusions were recruited into the study after obtaining informed written consent from parents or care givers. Patients who required at least six transfusions per year during past 2-years were considered as having transfusion dependent thalassaemia. Children with infective or inflammatory conditions and those who have undergone bone marrow transplantation were excluded from the study. Data were collected using an interviewer-administered pre-tested questionnaire by face to face interviews and perusing clinic records. Response rate was 100%. Questionnaire comprised of questions to obtain following information by interviewing patients; socio-demographic background, parental education level, family income and previous and current medical history. Following data were obtained by perusing clinical records; age at diagnosis, duration of transfusion, transfusion frequency, anthropometric data, serum ferritin levels, iron chelator medication and complication related to iron overload.

Data were analyzed using SPSS version 16.0. Categorical variables were expressed as counts and percentages and compared using χ2-test and odds ratios whereas means of continuous variables were compared using independent sample Student’s *t* test. p < 0.05 is considered as statistically significant. Ethical approval was obtained from Ethics Review Committee of Faculty of Medicine, University of Kelaniya.

### Results

#### Clinical and socio-demographic characteristics

Out of 54 children with transfusion dependent thalassaemia, 28 (51.9%) were males (Additional file 1: Table S1). Majority were aged between 11 and 16 years (57.4%) and were diagnosed before the age of 1 year (79.6%). The specific diagnoses were; β-thalassaemia major—45 (83.3%), HbE β-thalassaemia—7 (13.0%), Sickle β-thalassaemia—1 (1.8%) and heterozygous β-thalassaemia with triplicated α-globin genes—1 (1.8%). Majority (90.7%) of patients required blood transfusions every 3–4 weekly.

In a majority (79.6%) caregiver was the mother. Most (85.2%) patients were Sinhalese whilst, 5.6% were Tamils and 9.3% were Muslims. Majority of mothers were educated up to ordinary level (44.4%) whereas 22.2% were educated up to Advanced level. Majority of fathers were skilled labourers. In majority (57.4%) of the families, monthly income was above LKR 25,000.

#### Current status and trends of iron overload

**Fig. 1:**
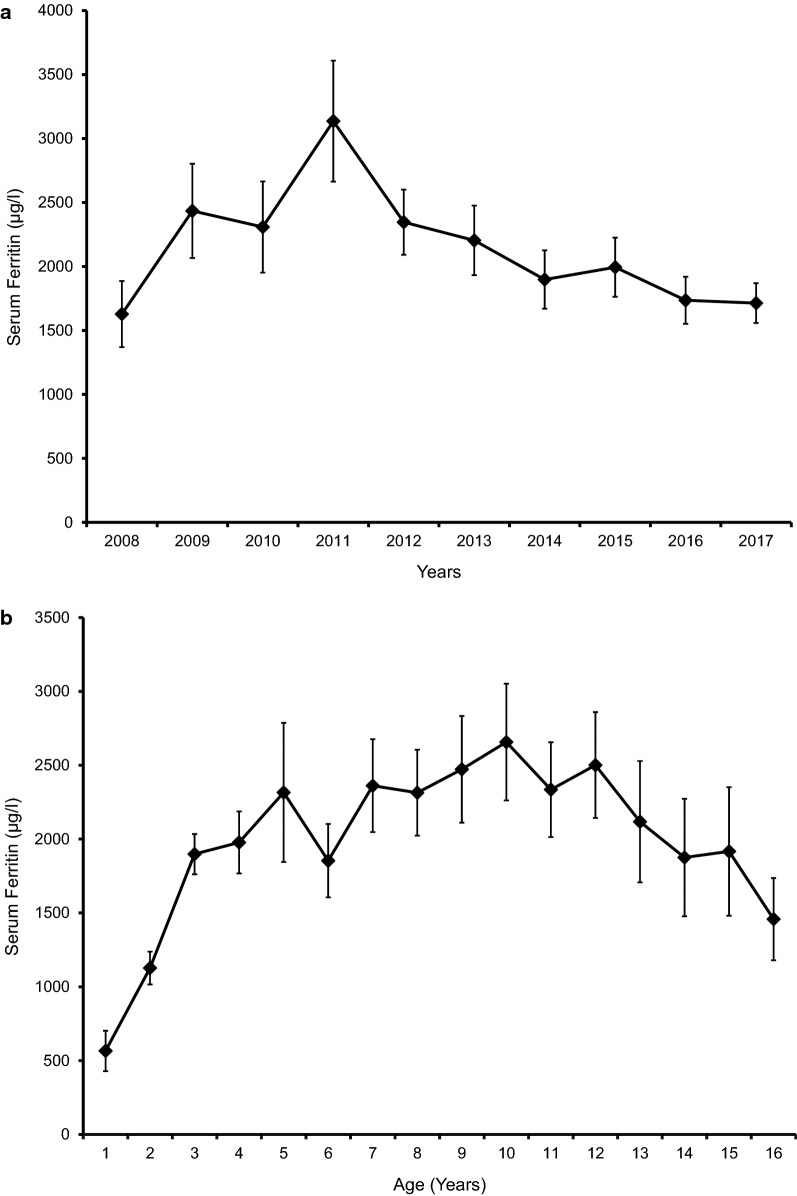
**a** Trends of serum ferritin over past 10 years. **b** Trend of life-time serum ferritin of the study population plotted longitudinally at yearly intervals. Mean serum ferritin values are plotted; error bars represent standard error of mean

Mean serum ferritin of the study population was 1778(± 1458) µg/l. Sixteen (29.6%) children had serum ferritin levels below 1000 µg/l and a majority (50%) had serum ferritin between 1000 and 2499 µg/l. In 2 (3.7%) children serum ferritin was above 5000 µg/l (Table. 1). Trend of mean serum ferritin over the past 10 years revealed steady and gradual decline between 2011 and 2017 (Fig. 1a). Trend of mean life-time serum ferritin of the study population plotted longitudinally at yearly intervals showed gradual rise until 5 years and plateauing thereafter (Fig. 1b). All except two patients were receiving iron chelator medication of which the most commonly used was monotherapy with oral deferasirox (61.1%). Seventeen (31.5%) patients were receiving combination of deferasirox and deferoxamine (Table. 1).

**Table 1:** Body iron status and iron chelator medication

	Frequency [N = 54]	Percentage (%)
Serum ferritin (most recent)		
< 1000 µg/l	16	29.6
1000–2499 µg/l	27	50.0
2500–4999 µg/l	9	16.7
> 4999 µg/l	2	3.7
Iron chelator medication		
Deferasirox	33	61.1
Deferoxamine	2	3.7
Deferasirox + deferoxamine	17	31.5
Not on chelation	2	3.7

#### Complications of iron overload

Screening for complications of iron overload revealed, 1 (1.8%) patient with evidence of cardiomyopathy in echocardiography. None of the patients had hypothyroidism, hypoparathyroidism or diabetes mellitus. Liver was ultrasonically normal in all patients however, 13 (24%) had elevated alanine aminotransferase (ALT). Thirteen (24.1%) patients had short stature and nine (42.8%) out of 21 patients aged 14 years or above had pubertal delay.

#### Determinants of optimal body iron control

Sixteen (29.6%) children had optimal (< 1000 µg/l) serum ferritin levels. Children with serum ferritin < 1000 µg/l were diagnosed with thalassaemia at a later age compared to others; 23.6(± 38.3) months vs 9.0(± 10.8) months (t = 2.15, p < 0.05). Importantly, there was no statistically significant differences in mean current age between children with optimal (11.4 ± 5.6 years) or high (10.6 ± 4.1 years) serum ferritin (t = 0.62, p = 0.53). Similarly, no difference was observed in the mean duration of transfusion in optimal (8.8 ± 5.9 years) or high serum ferritin (9.7 ± 4.2 years) groups (t = 0.63, p = 0.52). Associations between optimal body iron control and socio-demographic factors are shown in Table. 2.

**Table 2:** Association between socio-demographic factors and optimal body iron control

Socio-demographic factor	Number (%) of children with Serum ferritin < 1000 µg/l with associated factor (n = 16)	Number (%) of children with Serum ferritin > 1000 µg/l with associated factor (n = 38)	Odds ratio (95% CI)	χ2 value	p value
Male sex	10 (62.5%)	18 (47.4%)	1.85 (0.56–6.12)	χ2 = 1.03	0.37
Maternal education level-above grade 10	13 (81.3%)	27 (73.0%)	1.60 (0.37–6.84)	χ2 = 0.41	0.52
Family Income > LKR 25 000	13 (81.3%)	18 (47.4%)	4.81 (1.17–19.67)	χ2 = 5.28	< 0.05
Maternal employment	4 (25%)	14 (36.8%)	0.57 (0.15–2.11)	χ2 = 0.71	0.53

### Discussion

Transfusion-related iron overload is the primary cause of morbidity and mortality in patients with transfusion dependent thalassaemia [9]. There are three iron chelators—deferoxamine, deferasirox and deferiprone—currently available to manage iron overload however, none is efficacious enough to achieve a normal iron balance [10, 11]. Therefore, iron overload continues to be a major challenge in patients with β-thalassaemia.

Several methods are available to assess body iron status depending on the organs involved. Liver iron concentration is measured by magnetic resonance imaging (MRI) (T2* or R2), superconducting quantum interference device (SQUID) or biopsy whereas, cardiac iron is assessed by T2* MRI [12]. These methods have higher sensitivities and specificities however, are not freely available in resource poor countries [13, 14]. Serum ferritin is a reliable alternative and is recommended to assess body iron status in patients with thalassaemia worldwide [13]. In this study we have used serum ferritin as the indicator of iron status as it is the only investigation routinely available to patients in Sri Lanka [15].

The mean serum ferritin level of our patient cohort was 1778 µg/l which is considerably lower than the value (2992 µg/l) reported in a recent study done among forty patients in another centre in Sri Lanka [16]. Similarly, two recent studies done in India [17] and Pakistan [18] reported very high mean serum ferritin values, 2767 and 4236 µg/l, respectively among patients with thalassaemia. An audit done in 2010 in another centre of Sri Lanka noted that almost 50% of patients with β-thalassaemia major had serum ferritin above 2500 µg/l [19]. This was much higher than what we report; in our cohort only 20% had serum ferritin above 2500 µg/l. These findings suggest that degree of iron overload is much less in our cohort compared to other local and regional centres. This could be due to availability of iron chelating medication for free, improved compliance and high level of awareness among care givers regarding the disease and related complications.

Another striking observation of our study is the plateauing of serum ferritin levels from 5 years onwards. Similarly, we did not observe positive associations between ages of children or duration of blood transfusion with degree of body iron control. This is in contrast to the studies which report positive correlations between serum ferritin and duration of transfusion and age of the patients [16]. Also we observed a steady and gradual decline in the mean serum ferritin level between 2011 and 2018. Both these observations could be due to improved chelation regimens, increased compliance and availability of wider chelating options with the introduction of oral iron chelator deferasirox to the market. Additionally, we found that optimal body iron control is associated with later age at diagnosis and higher family income suggesting that patients belonging to higher social class are less likely to develop complications of iron overload. These findings are particularly important as several new therapies to cure thalassaemia are being currently evaluated and it is possible that a cure for this life-limiting disease is available to all patients within the next decade [20–23]. Therefore, it is vital that patients with thalassaemia have minimal iron overload and iron related complications to obtain maximum benefits from these novel therapies.

The most reported complications related to iron overload in our cohort were short stature and delayed puberty. None of the patients had hypothyroidism, hypoparathyroidism or diabetes mellitus. Conversely a Sri Lankan study done among 61 patients few years back reported 7 patients with cardiac failure, 2 with diabetes mellitus, 1 with hypoparathyroidism [19]. Hepatic alanine aminotransferase were elevated in 13 (24%) of our patients. Due to the facts that all these patients had normal liver architecture in ultrasonography, we believe that this is more likely to be due to an adverse effect of iron chelators medication rather than a complication of iron overload.

In conclusion our study reported lower iron overload compared to previous local and regional studies. Mean serum ferritin level gradually rose until 5-years of age and plateaued thereafter. Optimal body iron control was positively associated with older age at diagnosis of thalassemia and higher family income however, was not associated with the age or duration of transfusion.

## Limitations

One important limitation of this study is that it was performed only in a single centre. However, CNTH is one of the three main thalassaemia centres of the country and the results may be comparable in other centres of this part of the world. Secondly we used serum ferritin to assess iron overload which is known to produce falsely high values in the presence of infection or inflammation. Although some researches in the past have interpreted serum ferritin in combination of c-reactive protein levels, we did not perform c-reactive protein levels. Instead we screened patients at recruitment to exclude patients with infective and inflammatory conditions.

## Additional file

**Additional file 1: Table S1.** Clinical and socio-demographic characteristics of the study population.

## Abbreviations

RBC: red blood cells
CNTH: Colombo North Teaching Hospital
LKR: Sri Lankan Rupees
SQUID: superconducting quantum interference device
MRI: magnetic resonance imaging
