# Supplementary material for: Body iron status of children and adolescents with transfusion dependent β-thalassaemia: trends of serum ferritin and associations of optimal body iron control
Source: BMC Res Notes. 2018 Aug 2;11:547. doi: 10.1186/s13104-018-3634-9 (PMC6071405; doi:10.1186/s13104-018-3634-9)
Supplement: Supplementary file 1 — Additional file 1: Table S1. Clinical and socio-demographic characteristics of the study population. [file 13104_2018_3634_MOESM1_ESM.docx]

**Supplementary Table 1- Clinical and socio-demographic characteristics of the study population**

| **Characteristic** | **Frequency [N=54]** | **Percentage** |
| --- | --- | --- |
| Age distribution |  |  |
| < 2 years | 2 | 3.7% |
| 2 – 5 years | 5 | 9.3% |
| 6 – 10 years | 16 | 29.6% |
| 11 – 16 years | 31 | 57.4% |
|  |  |  |
| Sex distribution |  |  |
| Male | 28 | 51.9% |
| Female | 26 | 48.1% |
|  |  |  |
| Age at diagnosis |  |  |
| < 6 months | 21 | 38.9% |
| 6 – 11 months | 22 | 40.7% |
| 12 – 23 months | 4 | 7.4% |
| 24 – 59 months | 5 | 9.3% |
| 60 – 119 months | 1 | 1.9% |
| > 119 months | 1 | 1.9% |
|  |  |  |
| Thalassaemia type |  |  |
| β-thalassaemia major | 45 | 83.3% |
| HbE β-thalassaemia | 7 | 13.0% |
| Sickle β-thalassaemia | 1 | 1.9% |
| Heterozygous β-thalassaemia with triplicated α-globin genes | 1 | 1.9% |
|  |  |  |
| Transfusion frequency |  |  |
| < 3 weekly | 1 | 1.9% |
| 3 weekly | 24 | 44.4% |
| 4 weekly | 25 | 46.3% |
| 5-6 weekly | 1 | 1.9% |
| 7-8 weekly | 3 | 5.6% |
|  |  |  |
| Care giver^1^ |  |  |
| Mother | 43 | 79.6% |
| Father | 5 | 9.3% |
| Other | 3 | 5.6% |
|  |  |  |
| Ethnicity |  |  |
| Sinhala | 46 | 85.2% |
| Tamil | 3 | 5.6% |
| Muslim | 5 | 9.3% |
|  |  |  |
| Mother’s education level^2^ |  |  |
| None | 0 | 0 |
| Grade 1 -5 | 4 | 7.4% |
| Grade 6-10 | 9 | 16.7% |
| Ordinary Level | 24 | 44.4% |
| Advance Level | 12 | 22.2% |
| Higher education | 4 | 7.4% |
|  |  |  |
| Father’s occupation^3^ |  |  |
| Unemployed | 1 | 1.9% |
| Unskilled | 5 | 9.3% |
| Skilled | 24 | 50.0% |
| Lower Professional | 7 | 14.6% |
| Higher Professional | 9 | 18.8% |
|  |  |  |
| Monthly family income |  |  |
| < 10000 LKR | 2 | 3.7% |
| 10001 – 25000 LKR | 21 | 38.9% |
| 25001 – 50000 LKR | 25 | 46.3% |
| 50001 – 100000 LKR | 5 | 9.3% |
| > 100000 LKR | 1 | 1.9% |
|  |  |  |
| Number of children in the family |  |  |
| 1 | 14 | 25.9% |
| 2 | 28 | 51.9% |
| 3 | 11 | 20.4% |
| 4 | 0 | 0 |
| 5 | 1 | 1.9% |
|  |  |  |
| Number of Siblings with thalassaemia |  |  |
| 0 | 42 | 77.8% |
| 1 | 12 | 22.2% |

^1^ Three patients attended hospital on their own; ^2^ Mother of one patient was dead;

^3^ Two fathers were priests; Six did not provide information
